# Stock Returns, weather, and air conditioning

**DOI:** 10.1371/journal.pone.0219439

**Published:** 2019-07-05

**Authors:** Jie Hou, Wendong Shi, Jingwei Sun

**Affiliations:** 1 International School of Economics and Management, Capital University of Economics and Business, Beijing, China; 2 School of Economics, Renmin University of China, Beijing, China; 3 School of Economics, Central University of Finance and Economics, Beijing, China; Vilnius University, LITHUANIA

## Abstract

This study investigates the relationship between stock returns and local weather through a new channel—the influence of the air-cooling system installed in the New York Stock Exchange (NYSE). To our knowledge, we are the first to employ the use of air conditioning to examine whether and how weather, especially excessively high temperature, and other factors affect stock returns. Using data for 1885–1914, we show that lower Dow Jones Average (DJA) returns were significantly associated with hotness before the NYSE trading rooms were equipped with the cooling system in 1903, whereas this correlation is largely weakened afterward. We also find that before the introduction of the air-cooling system, the negative effect of high temperatures on stock returns was stronger when the precipitation was lower. We obtain consistent results when controlling for the calendar anomalies such as the May-to-October effect, the Monday effect, and the effect of macroeconomic conditions.

## Introduction

The linkage between weather and stock market returns has been long documented, especially in the literature on finance and psychology. The correlation is generally interpreted via the negative mood effect of bad weather, such as extreme temperature, strong wind, and lack of sunshine [1][2]. While explaining stock returns through a sentimental way and relating price-setting to Mother Nature are intuitively appealing, many researchers suspect that stock prices are not systematically affected by the local weather and that the changes in stock returns might simply be explained by seasonal anomalies such as the “Sell in May and go away” behavior [3].

This paper re-visits the presence of weather’s mood effect on the stock returns from a new perspective—the influence of the air-cooling system installed in the New York Stock Exchange (NYSE). The invention of air conditioning, more specifically, the cooling system designed by Alfred Wolff, provides a valuable experiment to further investigate the mysterious mood effects of weather. In 1899, Alfred Wolff used a refrigeration unit to circulate a brine solution through pipes and blow air to cool the room, which was considered as the beginning of modern air conditioning. In 1902, the new NYSE building was equipped with a 300-ton central cooling system designed by Alfred Wolff and became one of the first facilities in the world to have air conditioning. On April 22, 1903, the NYSE moved into this new building and the traders started to work in the air-conditioned environment. Since the cooling system helped keep the temperature in the trading rooms at more pleasant levels while it was hot outside, we suspect that the effects of the weather on the traders’ performances (if there were any) changed after the installation of air conditioning. Our hypothesis is that if weather, especially the excessively high temperature, affects stock returns through traders’ moods, then this effect should be weaker after the installation of the air conditioning system because the traders were better protected against the hotness by the cooling system.

Using daily weather data for New York City and the daily indices of the Dow Jones Average (DJA) from 1885 to 1914, we find that the stock return was significantly negatively correlated with the excessively high temperature before the installation of the cooling system, whereas this correlation was insignificant thereafter. When we take precipitation into consideration, we find that before air conditioning was introduced to the NYSE, the high temperature had a greater effect on returns on days with low precipitation, while the influence of excessively high temperature was reduced when the precipitation was high. Furthermore, we control for other effects that might cause changes in stock returns. After considering the calendar anomalies, such as the May to October effect, the Monday effect, and the macroeconomic conditions, we obtain consistent results on the diminishing mood effect of temperature after the cooling system was installed. Our results further verify the weather’s mood effect on the traders’ behavior as documented in the previous studies [1][2].

The remainder of the paper is organized as follows. Section two presents the background and literature review. Section three describes the data used for empirical analyses. Section four outlines the methodology and discusses the results. Section five concludes.

## Background

### The mood effect of the weather

Weather’s effect on human’s mood and behavior has been widely discussed in psychology literature since decades ago. Although the causality seems to be straightforward, the empirical studies produce mixed results that either finds a strong correlation between weather and psychological changes or claims weather has little effect [4][5]. Among the empirical evidences on weather’s effect, some indicate that temperature has a significant influence. In particular, Rotton and Frey [6] argue that family disturbances and assaults against persons are positively correlated with daily temperatures. Anderson and Craig [7] show that heat increases aggression such as violent crime and spouse abuse. Keller et al. [8] find that pleasant temperature and barometric pressure is related to higher mood and better memory, whereas hotter weather is associated with the lower mood in the summer. Trading activities, as commonly considered, highly correlate to traders’ skills, personalities and moods; meanwhile, the outcomes can be directly and immediately presented by stock returns. Therefore, the stock market provides researchers with a good opportunity to examine the above causality.

There are numerous arguments to support that local weather plays an important role in traders’ moods and behaviors and therefore influences stock prices. For instance, Saunders [1] first shows that the cloud cover in New York City is correlated to the DJIA and NYSE/AMEX return index. Using data for 26 stock exchanges in the world, Hirshleifer and Shumway [9] find that morning sunshine is strongly significantly associated with daily stock returns, and the effects of sunshine dominate those of rain and snow. Kamstra et al. [10] claim that stock returns are linked to the changes of investors’ moods caused by changes of the season. Keef and Roush [11] find that the returns of Australian stock indices are negatively influenced by the temperature in Sydney. Perhaps the closest to our study is Cao and Wei [2], who use nine international stock indices including the US equal-weighted and the value-weighted indices. They find a significant negative correlation between temperature and stock returns using the equal-weighted indices regardless of calendar anomalies, whereas the correlation is weakened for the value-weighted indices with controlling for seasonal dummies.

There are also studies showing none or mild impacts of weather on stock returns. For instance, Theissen [12] finds that German private investors make predictions regardless of differences in temperature. Jacobsen and Marquering [3] claim that the seasonal volatility of stock returns could not be explained by investors’ mood changes caused by cloudiness or temperature variations. When controlling for the calendar anomalies such as the May to October effect and the Monday effect, the temperature’s effect on stock returns is insignificant. Therefore, to examine the significance of the temperature’s effect, we control for the calendar anomalies as well as changes in macroeconomic conditions.

### The January effect, the May to October effect, and the October effect

The January effect (or the so-called turn-of-the-year effect) is related to the fact that stock returns in January are higher than those in other months; the May to October (MTO) effect suggests lower returns during the period from May to October; the October effect refers to lower returns in October. Such seasonality of stock returns is discussed in many studies, such as Cadsby [13], Ariel [14], Schwert [15], Jacobsen and Marquering [3], and Levy and Yagil [16]. Studies that support the significance of calendar anomalies tend to interpret the seasonality of stock returns in terms of the holiday behavior of investors. For instance, Hong and Yu [17] find that the MTO effect is stronger in the countries farther away from the equator where vacations are usually in summer. They claim that trading activity falls because the investors are “gone fishing” during the summer vacation and the mean returns are lower. However, most of these studies omit the possible influence of weather, such as excessively high temperatures and lack of sunshine, on traders’ moods and performance.

### The Monday effects

There are substantial studies documenting significantly low stock returns on Mondays, which is observed in many countries in the world [18][19]. This Monday anomaly is also known as the weekend effect, the day-of-the-week effect, or the turn-of-the-week effect. There are various potential explanations for the cause of the Monday effect. For instance, Damodaran [20] attributes the Monday anomaly to the timing of corporate releases after Friday’s close. Lakonishok and Maberly [21] and Chen et al. [22] find that fewer institutional trading and more individual trading happen on Mondays and conclude that trading by the less sophisticated individual investors is related to the negative returns. Chen and Singal [23] argue that the short sellers are likely to close their speculative positions on Fridays and reestablish new short positions on Mondays, which causes stock prices to fall on Mondays.

## Data

Our data set covers a long period from February 17, 1885 to July 31, 1914, including the year 1903 when the NYSE started to use the cooling system in the trading rooms. More recent data are accessible, but we do not extend our data to the present to exclude the impacts of World War I (1914–1918) as well as to keep a more reasonable time span (and amount of observations) to present the influence of air conditioning. According to the historical record of the NYSE, the new NYSE building was equipped with the cooling system and put into use on April 22, 1903. Therefore, the pre-cooling period is from February 17, 1885 to April 21, 1903 and the post-cooling period is from April 22, 1903 to July 30, 1914.

In order to check the effects of weather on stock returns before and after the installation of the air conditioning system, we collect data for the daily returns on the indices of the DJA as well as a set of weather variables for New York City (Central Park observation tower). The historical DJA indices were obtained from Williamson [24]. Here, we measure the stock returns using the DJA indices instead of the S&P 500 indices because the DJA, as the first stock market index, was published in 1885, whereas the S&P 500 was not introduced until 1923. The weather data were collected from the National Climactic Data Center (www.ncdc.noaa.gov). Since the data we need are dated about one hundred years back, available information on weather is not as detailed as that for the more recent years. For example, we do not have information on cloudiness or humidity. Hence, we mainly use temperature and precipitation to indicate the weather condition. We collect daily data on maximum temperature (TMAX, tenths of degrees C) and precipitation (PREC, tenths of mm).

Furthermore, we control for calendar anomalies by including an MTO dummy (MTO) and a Monday dummy (MON). The January effect is not included here, because we aim at investigating the effect of excessive heat on stock returns and temperatures in New York City are generally low in January. Changes in macroeconomic conditions are also considered. We include a macroeconomic dummy (ECON) in the model to indicate the stock market environment (bull or bear market) and the macroeconomic situation.

Table 1 presents a summary of statistics. For the whole period, about 60.14% of the sample is considered as a bull market. This ratio barely changes across the pre- and post-cooling periods.

**Table 1:** Summary statistics.

	02/17/1885–07/31/1914			02/17/1885–04/21/1903			04/22/1903–07/31/1914		
	Mean	Std. Dev.	N	Mean	Std. Dev.	N	Mean	Std. Dev.	N
**Stock Return**	0.0207	0.9199	2948	0.0302	0.9578	1785	0.0061	0.8588	1163
**Highest Temperature**	27.1918	3.2996	2948	27.2496	3.3489	1785	27.1029	3.2220	1163
**Precipitation**	28.1381	87.7578	2948	30.3423	90.9748	1785	24.7549	82.5008	1163
**Bull**	0.6014	0.4897	2948	0.6084	0.4882	1785	0.5907	0.4919	1163

Stock return is the daily return for the DJA index. Daily highest temperature is measured at tenths of degrees C. Daily precipitation is measured at tenths of mm. BULL is equal to 1 if it was in a bull market and equals 0 otherwise.

**Fig 1:**
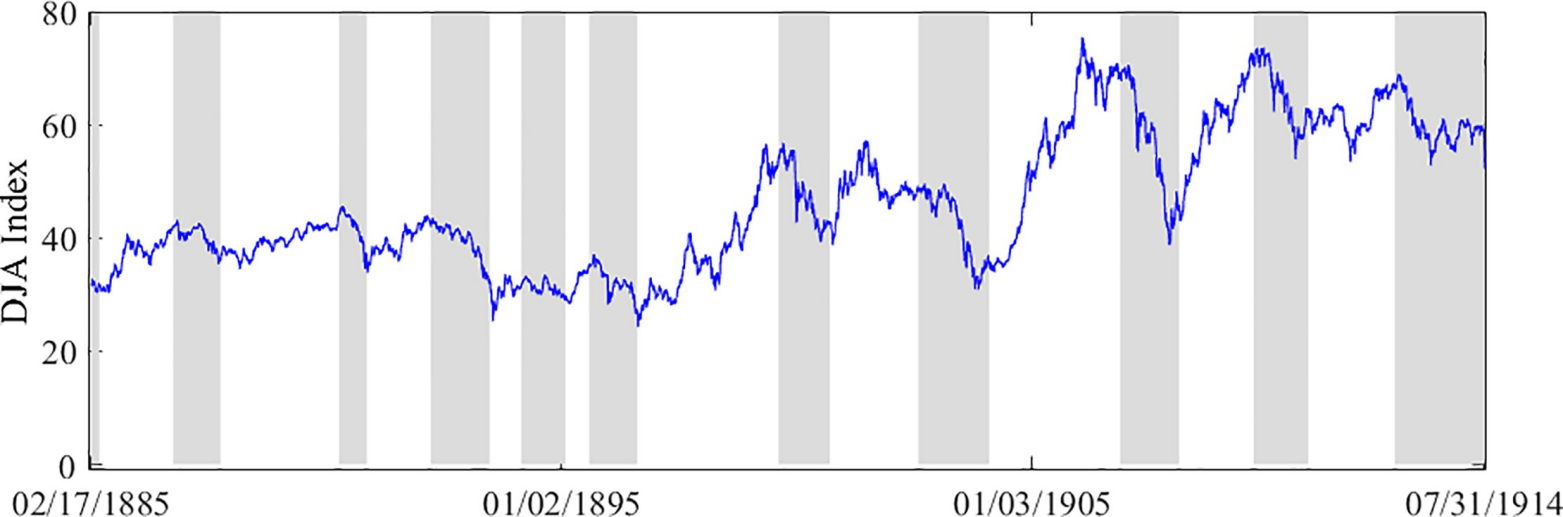
DJA index.

A detailed description of bull/bear market regimes is shown in Fig 1. Fig 1 plots the raw DJA indices. The shaded regions indicate the bear market periods, which are time spans between two consecutive bull markets. Bull market regimes are determined according to the stock market turning points proposed by Gonzalez et al. [25].

## Methodology and empirical results

### Testing the mood effect before/after installation of the cooling system

In order to examine the changes in the relationship between stock returns and weather after air conditioning was introduced to the NYSE, we consider the differences in stock returns under comfortable temperatures and excessively high temperatures. 22°C is believed to be the most comfortable environment temperature for human beings. For example, Seppanen et al. [26] show that workers’ performance increases with temperatures up to 21–22°C and decreases with temperatures above 23–24°C; the highest productivity is at a temperature of around 22°C. At 30°C, the performance is only 91.1% of the maximum. Therefore, for both the pre-cooling and the post-cooling periods, we drop the matched data if the daily maximum temperature is lower than 22°C. We divide the remainder into two groups, “Group 0,” with a TMAX falling in the range of [22°C,30°C], and “Group 1,” with a TMAX higher than 30°C. Alternative cutoffs are adopted as a robust check and the results are consistent with those presented in this paper.

The daily return for the DJA index is computed as follows:

$$R_{t}=log(I_{t}/I_{t-1})\times 100$$ (1)

where *R*_*t*_ is the daily percentage return on the DJA index on day *t*, *I*_*t*_ and *I*_*t*−1_ are the closing values of the stock index on day *t* and day *t*−1, respectively.

To test whether the stock returns under excessively high temperatures and comfortable temperatures are significantly different, we use z-statistic, as in Saunders [1]:

$$t-statistics=\frac{\mu_{H}-\mu_{C}}{\sqrt{\sigma_{H}^{2}/n_{H}+\sigma_{C}^{2}/n_{C}}}$$ (2)

where μ and *σ*^2^ are the mean returns and the variance of returns, respectively. The subscripts are used to distinguish groups, i.e. “H” (hot) for Group 1 and “C” (comfortable) for Group 0. The test is performed for the pre-cooling period (02/17/1885–04/21/1903), the post-cooling period (04/22/1903–07/31/1914), as well as the whole time period. Corresponding results are presented in Table 2.

**Table 2:** Relation between temperature and stock returns.

	02/17/1885–07/31/1914			02/17/1885–04/21/1903			04/22/1903–07/31/1914		
	N	Mean	Std. Err.	N	Mean	Std. Err.	N	Mean	Std. Err.
**Group 0 (C)**	2394	0.0394	0.0186	1438	0.0563	0.0247	956	0.0139	0.0282
**Group 1 (H)**	554	-0.0598	0.0407	347	-0.0777	0.0559	207	-0.02975	0.0555
**Combined**	2948	0.0207	0.0169	1785	0.0302	0.0227	1163	0.0061	0.0252
**Difference**		0.0992***	0.0433		0.1340***	0.0572		0.0437	0.0659

The asterisk *** indicates statistical significance at the 1% level.

As shown in Table 1, returns for the two groups (H and C) are significantly different for the pre-cooling period (02/17/1885–04/21/1903); the difference for the whole time period our data covers (02/17/1885–07/31/1914) is weaker but still significant; when it comes to the post-cooling period (04/22/1903–07/31/1914), returns for the two groups do not significantly differ from each other. The results indicate a varying correlation between excessively high temperatures and NYSE stock returns before and after the installation of the air-cooling system. Stock returns can be affected by many factors and the correlation between weather and returns does not necessarily imply causation, especially when there might be bias caused by omitted variables. Since the relationship between returns and temperature changed with respect to the indoor comfort, one potential explanation to this changing linkage can be weather’s mode effect. In particular, the excessively high temperatures had a negative mood effect on the NYSE traders and lead to lower stock returns before the cooling system was introduced. After being protected by the cooling system, traders were able to partially get rid of the negative influence of the high temperatures on hot days and performed (almost) as well as they did on the comfortable days. Temperature’s effect during the pre-cooling period is so stupendous that it results in the significant difference between the two groups for the whole period. The results shown in Table 2 confirm our hypothesis that lower stock returns are associated with higher temperatures, but this correlation is weakened after introducing the air conditioning system to the stock exchange.

Furthermore, we consider the effect of daily precipitation and interact it with temperature. Therefore, the above two groups (H and C) can be further distinguished as four groups: HW (hot and wet), HD (hot and dry), CW (comfortable and wet), and CD (comfortable and dry). Here, we define “W” as precipitation above the mean precipitation and “D” as otherwise. We perform the t-statistic tests for the groups H and C under different levels of precipitation and report the results in Table 3.

**Table 3:** Relation between temperature and stock returns (for different precipitation levels).

	02/17/1885–07/31/1914		02/17/1885–04/21/1903		04/22/1903–07/31/1914	
	High Prec (W)	Low Prec (D)	High Prec (W)	Low Prec (D)	High Prec (W)	Low Prec (D)
**Group 0 (C)**	-0.0132	0.0504	-0.0153	0.0719	-0.0029	0.0174
**Group 1 (H)**	-0.0376	-0.0647	-0.0623	-0.0812	-0.0225	-0.0312
**Combined**	-0.0180	0.0289	-0.0247	0.0423	-0.0064	0.0087
**Difference**	0.0244	0.1151***	0.0471	0.1530***	0.0196	0.0486

The asterisk *** indicates statistical significance at 1% level.

According to Table 3, when the daily precipitation is low, returns under excessively high temperature are significantly lower than those under comfortable temperatures during the pre-cooling period (02/17/1885–04/21/1903), but this difference is largely reduced after the NYSE was equipped with air conditioning (04/22/1903–07/31/1914). When the precipitation is high, returns under high temperatures and comfortable temperatures do not differ significantly. Here, we use the information on precipitation which is quite different from humidity due to the availability of data. Intuitively, we consider high humidity to make people feel worse in a hot environment, but high precipitation does the opposite. The precipitation, in particular, summer rainfall, might give relief from hot weather by bringing fresh air, blocking sunshine, and lowering temperature. Before the cooling system was introduced, when the precipitation was low, excessively high temperatures had greater negative effects on traders’ moods and feelings and lead to worse performance in trading activities.

### Estimating the mood effect and the calendar anomalies

We consider the mood effect as well as the calendar anomalies in our model. In order to investigate the changes in the above effects after the air conditioning system was adopted in 1903, we distinguish data for pre-cooling and post-cooling periods in regression models. The following procedures are applied to both periods. To estimate the mood effect of weather, we look at both temperature and precipitation. Correlation between low productivity and excessive heat is documented in many studies [26][27]. Here, we estimate the following equation:

$$R_{t}=\beta_{1}R_{t-1}+\beta_{2}{TMAX}_{t}+\beta_{3}{PREC}_{t}+\varepsilon_{t}$$ (3)

where *R*_*t*_ is the daily percentage return on the DJA index on day *t*, *TMAX*_*t*_ is equal to 1 if the maximum temperature on day *t* is higher than 30°C, and it equals 0 otherwise *PREC*_*t*_ is 1 if the precipitation on day *t* is above the mean precipitation, and 0 otherwise, and *ε*_*t*_ is the error term. First-order lagged returns R_t−1_ are also included. Here, we consider the maximum temperature instead of the mean temperature because the cooling system mainly performed against high temperatures. Without access to the hourly temperature data, we use the maximum temperature instead of mean temperature to reflect the necessary use of the air conditioning.

We include dummies for the seasonal anomalies and bull/bear markets in the following equation:

$$R_{t}=\beta_{1}R_{t-1}+\beta_{2}{TMAX}_{t}+\beta_{3}{PREC}_{t}+\beta_{4}{MTO}_{t}+\beta_{5}{MON}_{t}+\beta_{6}{ECON}_{t}+\varepsilon_{t}$$ (4)

where MTO is equal to 1 if *t* was during the period from May to October; MTO is 0 otherwise. MON is equal to 1 if *t* was a Monday; otherwise, MON equals 0. ECON captures the economic environment to consider the seasonality of stock returns caused by the seasonal changes in macroeconomic conditions and business cycle [3]. Two measures are used for ECON: bull market (BULL) and GDP growth rate (GDP). BULL is equal to 1 if it was in a bull market and equals 0 otherwise. GDP is calculated by the percentage of GDP difference for two consecutive years. In line with the literature, the MTO dummy is included in the regression to eliminate the MTO anomaly [28]. MON is considered to control for the possible Monday effect [23][16]. Bull and bear markets are defined according to the stock market turning points proposed by Gonzalez et al. [25]. We do not include dummies to control for the January effect or for the October effect. Our study is focused on the influence of the excessively high temperature before and after the use of a cooling system, which is not feasible for New York City in January. The October anomaly is included in the MTO dummy. The results are reported in Table 4.

**Table 4:** Regression analysis.

	02/17/1885–07/31/1914				02/17/1885–04/21/1903				04/22/1903–07/31/1914			
	(1)	(2)	(3)	(4)	(1)	(2)	(3)	(4)	(1)	(2)	(3)	(4)
***R***_***t*−1**_	-0.0030 (-0.16)	-0.0037 (-0.20)	-0.0025 (-0.13)	-0.0038 (-0.20)	0.0072 (0.30)	0.0065 (0.27)	0.0079 (0.33)	0.0066 (0.28)	-0.0476 (-1.62)	-0.0476 (-1.62)	-0.0464 (-1.58)	-0.0475 (-1.62)
**TMAX**	-0.1151*** (-2.41)	-0.1098** (-2.29)	-0.1050** (-2.19)	-0.1098** (-2.29)	-0.1535*** (-2.42)	-0.1483*** (-2.33)	-0.1395** (-2.19)	-0.1479*** (-2.32)	-0.0596 (-0.83)	-0.0553 (-0.77)	-0.0583 (-0.81)	-0.0569 (-0.79)
**PREC**	-0.0637 (-1.28)	-0.0620 (-1.25)	-0.0611 (-1.23)	-0.0620 (-1.25)	-0.0857 (-1.31)	-0.0872 (-1.33)	-0.0842 (-1.29)	-0.0868 (-1.32)	-0.0351 (-0.47)	-0.0348 (-0.46)	-0.0373 (-0.49)	-0.0371 (-0.49)
TMAX*PREC	0.0908 (0.80)	0.0893 (0.79)	0.0781 (0.69)	0.0893 (0.79)	0.1067 (0.73)	0.1079 (0.74)	0.0971 (0.66)	0.1081 (0.74)	0.0971 (0.55)	0.1006 (0.56)	0.0883 (0.50)	0.1001 (0.56)
**MON**		-0.0243 (-0.52)	-0.0267 (-0.58)	-0.0242 (-0.52)		-0.0907 (-1.46)	-0.0909 (-1.46)	-0.0895 (-1.44)		0.0668 (0.98)	0.0615 (0.90)	0.0664 (0.97)
**MTO**		-0.1540 (-1.52)	-0.1637 (-1.61)	-0.1544 (-1.52)		-0.1858 (-1.31)	-0.1878 (-1.33)	-0.1907 (-1.34)		-0.1919 (-1.33)	-0.2093 (-1.46)	-0.1911 (-1.33)
**BULL**			0.1092*** (3.16)				0.0972** (2.09)				0.1151*** (2.25)	
**GDP**				0.0004 (0.11)				0.0043 (0.92)				-0.0046 (-1.07)
**Constant**	0.0505*** (2.44)	0.2027** (2.02)	0.1460 (1.44)	0.2017** (2.01)	0.0716*** (2.56)	0.2661 (1.89)	0.2070 (1.45)	0.2523 (1.79)	0.0217 (0.72)	0.1957 (1.38)	0.1467 (1.03)	0.2046 (1.45)
**N**	2948	2948	2948	2948	1785	1785	1785	1785	1163	1163	1163	1163
**R**^**2**^	0.0024	0.0032	0.0066	0.0032	0.0041	0.0062	0.0087	0.0067	0.0029	0.0052	0.0096	0.0062
**Durbin-Watson Test**	2.002	2.002	2.002	2.002	2.002	2.002	1.999	2.002	1.993	1.9930	1.989	1.9942

Numbers in parentheses are t values, which are based on heteroscedasticity consistent standard errors.

The asterisks **, and *** indicate statistical significance at the 5% and 1% levels, respectively.

According to Table 4, lower returns are significantly associated with higher temperature for the whole period, whereas other factors except the bull market have minor effects on stock returns. For the pre-cooling period, the negative correlation between daily return and temperature is more significant, implying a stronger impact of the excessively high temperature on returns. The effect of hotness is greatly reduced in the post-cooling period when the indoor temperature was controlled using the air conditioning system. The results confirm our previous findings shown in Table 3. In addition, the Durbin-Watson diagnostic test is performed to determine whether the error term in our regression model has a serial correlation. The values of d are very close to 2, suggesting no evidence for autocorrelation.

## Conclusions

It is widely documented that weather influences people’s moods and therefore affects their work performance and productivity. Among all the weather factors, the temperature is given great attention and generally believed to be negatively correlated with productivity [26]. Previous studies on the financial market find that weather has an impact on stock returns through its mood effect on the traders. Especially, lower returns are associated with higher temperatures [1][2]. However, the soundness of this correlation is questioned by other studies in the sense that the significance of temperature anomalies sometimes can be absorbed by seasonal anomalies [3].

In this study, we attempt to examine the relationship between temperature and stock returns through a new channel—the use of air conditioning. Starting from April 22, 1903, traders in the NYSE were provided with comfortable cooling by the air conditioning system. Therefore, our hypothesis is that excessively high temperatures yield low stock returns, whereas this correlation is weakened after the introduction of the cooling system.

Using daily temperature data and DJA returns for 1885–1914, we show that stock returns under comfortable temperatures are significantly higher than those under excessively high temperatures during the pre-cooling period (1885–1903), whereas the difference is insignificant for the post-cooling period (1903–1914). The estimated results further confirm this finding and reports a significantly negative correlation between returns and temperature for the pre-cooling period as well as an insignificant correlation for the post-cooling period. Furthermore, we control for calendar anomalies such as the MTO effect and the Monday effect as a robustness check. Our estimates with calendar anomalies are consistent with the previous results.
